# Systemic inflammation induces acute working memory deficits in the primed brain: relevance for delirium

**DOI:** 10.1016/j.neurobiolaging.2010.04.002

**Published:** 2012-03

**Authors:** Carol Murray, David J. Sanderson, Chris Barkus, Robert M.J. Deacon, J. Nicholas P. Rawlins, David M. Bannerman, Colm Cunningham

**Affiliations:** a Trinity College Institute of Neuroscience, School of Biochemistry & Immunology, Trinity College Dublin, Dublin, Ireland; b Department of Experimental Psychology, South Parks Road, University of Oxford, Oxford, United Kingdom

**Keywords:** Microglia, Delirium, Dementia, Priming, Synaptic, Predisposition, Infection, Inflammation, Animal model

## Abstract

Delirium is an acute, severe neuropsychiatric syndrome, characterized by cognitive deficits, that is highly prevalent in aging and dementia and is frequently precipitated by peripheral infections. Delirium is poorly understood and the lack of biologically relevant animal models has limited basic research. Here we hypothesized that synaptic loss and accompanying microglial priming during chronic neurodegeneration in the ME7 mouse model of prion disease predisposes these animals to acute dysfunction in the region of prior pathology upon systemic inflammatory activation. Lipopolysaccharide (LPS; 100 μg/kg) induced acute and transient working memory deficits in ME7 animals on a novel T-maze task, but did not do so in normal animals. LPS-treated ME7 animals showed heightened and prolonged transcription of inflammatory mediators in the central nervous system (CNS), compared with LPS-treated normal animals, despite having equivalent levels of circulating cytokines. The demonstration that prior synaptic loss and microglial priming are predisposing factors for acute cognitive impairments induced by systemic inflammation suggests an important animal model with which to study aspects of delirium during dementia.

## 1 Introduction

Delirium is an acute and transient impairment of global cognitive function, causing marked impairments in consciousness, attention, immediate recall, orientation (temporal and spatial), and perception (Burns et al., 2004; Meagher, 2009). It is highly prevalent in the aged and demented population and it is now well accepted that episodes of delirium hasten cognitive and functional decline and increase mortality (Fick et al., 2002; MacLullich et al., 2009; McCusker et al., 2001; Murray et al., 1993; Pitkala et al., 2005; Rahkonen et al., 2000; Rockwood et al., 1999). Recent high profile studies have reiterated this with a specific focus on patients with Alzheimer's disease (Fong et al., 2009b). Despite these important economic and medical imperatives, delirium is understudied and poorly understood (Fong et al., 2009a). One reason for this is the lack of biologically relevant animal models.

It is well accepted that systemic inflammation, caused by infection, surgery, or injury can induce episodes of delirium in elderly or demented patients (Beloosesky et al., 2007; Elie et al., 1998; Lemstra et al., 2008; Lerner et al., 1997; van Munster et al., 2008) while much less commonly causing similar dysfunction in younger or nondemented patients. The mechanisms of this dysfunction remain unclear. We have previously shown that microglia, the major macrophage population of the brain, are primed by prior neurodegenerative pathology to respond more robustly to systemic inflammatory signals (Cunningham et al., 2005). Here, we hypothesized that systemic inflammation, induced in animals with early stage neurodegeneration (ME7 prion disease), characterized by synaptic loss and primed microglia in the hippocampus, would induce acute onset and transient working memory dysfunction that is not induced by similar challenges in normal animals. In previous studies we reported a systemic inflammation-induced learning deficit in a reference memory task (Cunningham et al., 2009a) but novel approaches were necessary to investigate whether prior hippocampal pathology would be sufficient to predispose animals to systemic inflammation-induced working memory deficits that were acute onset and transient. These are key criteria in the clinical diagnosis of delirium.

ME7 is a prion disease strain that has been mouse-adapted and constitutes a real progressing neurodegenerative disease, characterized by extracellular amyloidosis, synaptic loss, robust neurodegeneration, and progressive cognitive decline (Betmouni et al., 1999; Cunningham et al., 2003). Because the early pathology is chiefly hippocampal, disease-associated cognitive impairments consist of failure on spatial working and reference memory tasks that, in the mouse, are hippocampal-dependent. We predicted that acute systemic inflammation would induce hippocampal-dependent working memory impairments at a time when such impairments were not yet present in disease per se. Because systemic inflammation causes changes in appetite, locomotor activity, motivation, and stress, which can confound many commonly used tests of hippocampal function in mice (Cunningham and Sanderson, 2008), we have designed and validated a novel shallow water T-maze alternation task that is less sensitive to these confounding factors. We challenged prion-diseased (ME7) and normal mice, at 12 weeks postinoculation, with bacterial endotoxin at 100 μg/kg and analyzed the acute effects (3–8 hours) on working memory. We also assessed learning in an egocentric Y-maze task to assess the generality of performance deficits. In order to assess the degree of, and possible mechanisms underpinning, the primed brain's heightened responses to systemic inflammation, we examined a number of inflammatory receptors and assessed the time course of expression of systemic and central nervous system (CNS) cytokines and effector molecules. Our findings suggest a model system that will be important in delineating the systemic and central inflammatory contributions to acute cognitive dysfunction, as seen in delirium.

## 2 Methods

### 2.1 Animals and stereotaxic surgery

Female C57BL/6 mice at 8–10 weeks of age (Harlan Olac Ltd, Bicester, United Kingdom) were housed in cages of 5 at 21 °C with a 12 : 12 hour light-dark cycle with food and water ad libitum. They were anesthetized with intraperitoneal Avertin (Sigma, Poole, UK) and stereotactically injected with 1 μl of a 10% w/v scrapie (ME7 strain)-infected C57BL/6 brain homogenate (or 10% w/v normal brain homogenate (NBH): at bregma: anterior-posterior −2.5 mm, lateral −1.7 mm, depth −1.6 mm) using a Hamilton microsyringe (Sigma, Poole, UK). Additional animals (*n* = 8) were injected intracerebrally (4 injections bilaterally) with N-methyl-D-aspartic acid (NMDA; 10 mg/mL) to ablate the hippocampus under isoflurane anesthesia (approximately 2%) with perisurgical analgesia (carprophen 5 mg/kg). Chlordiazepoxide (CDZP; 10 mg/kg) and atropine (0.075 mg/kg) were given to minimize seizure activity and bronchial secretions respectively. In all other respects the hippocampal lesions were performed as previously described (Deacon et al., 2002). Sham-operated animals (*n* = 12) had 8 holes drilled in the skull, but no intracerebral injections were made. All animal procedures were done in strict accordance with UK Home Office license and Irish Department of Health regulations.

### 2.2 Intraperitoneal challenges

Experimental groups with ME7 or NBH at 12 weeks postinoculation were injected intraperitoneally (i.p.) with 100 μg/kg (or 200 μg/kg to age-matched naive mice) of lipopolysaccharide (LPS; equine abortus, Sigma L5886, Poole, UK) in a volume of 200 μl saline. This dose mimics a mild infection, producing small changes (< 1 °C) in core body temperature. Controls were administered 200 μl nonpyrogenic saline in each case.

### 2.3 T-maze alternation: working memory

**Figure S1:**
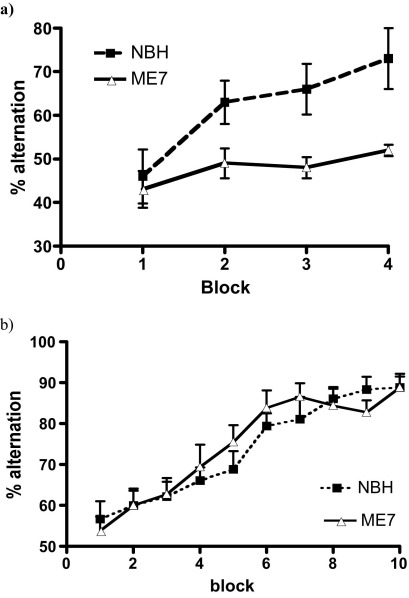
Rate of learning of correct responding (alternation) in the shallow water T-maze in ME7 versus NBH animals late or early in disease. a) Alternation performance of ME7 and NBH animals was examined daily starting from 18 weeks post-inoculation for four successive days. b) Alternation performance of ME7 and NBH animals was examined daily starting from 10 weeks post-inoculation for 10 successive days. Data are shown as mean ± SEM and were analysed by repeated measures ANOVA using training block as the within subjects factor and disease as the between subjects factor. n = 10 for ME7 and n = 9 for NBH at 18 weeks and n = 20 for ME7 and n = 15 for NBH at 12 weeks.

We assessed hippocampal-dependent working memory (3–8 hours post-LPS) using alternation behavior in a novel “escape from shallow water” T-maze task to allow assessment of performance in animals experiencing sickness behavior. The T-maze was constructed of black Perspex with dimensions (cm): long axis 67, short axis 38, depth 20, and arm width 7. There was a single 40 mm diameter hole at the end of each choice arm, 2 cm from the floor. Black exit tubes were inserted into these holes (these may also be blocked to prevent exit). A “guillotine” door was inserted to prevent access to 1 or other choice arm. This maze was filled with water at 20 °C to a depth of 2 cm to motivate mice to leave the maze by “paddling” or walking “on tip-toe” to an exit tube. Animals were taken with their cage mates to a holding cage. Each mouse was placed in the start arm of the maze with 1 arm blocked such that they were forced to make a left (or right) turn, selected in a pseudorandom sequence (equal numbers of left and right turns, no more than 2 consecutive runs to the same arm). On making this turn the mouse could escape from the water by entering the small tube, and then a transit tube, in which it was carried to another holding cage. The mouse was held here for 25 seconds (intratrial interval) during which time the guillotine door was removed and the exit tube was switched to the alternate arm. The mouse was then replaced in the start arm and could choose either arm. The mouse must alternate from its original turn to escape. On choosing correctly mice escape to the transit tube as before and are returned to their home cage. On choosing incorrectly the mice were allowed to self-correct to find the correct exit arm. Validation of this task is shown in supplementary data (Figure S1).

### 2.4 Y-maze egocentric reference memory

The egocentric Y-maze was identical to that used in a previous study (Cunningham et al., 2009a) with the exception that it was surrounded with black plastic material to a height of >1 meter such that visual cues were negated as completely as possible (as opposed to the use of visual cues in the allocentric version). Mice were placed in the start arm and both this arm and the exit arm were fixed for any given animal. The animal, thus, had to learn the correct body turn to exit the maze. The start arm/exit arms were counterbalanced across groups. ME7 or NBH-injected mice, with or without LPS were placed in this maze 3 hours posttreatment for 12 trials and the numbers of incorrect trials were recorded. Probe trials were conducted to confirm egocentric strategy.

### 2.5 Tissue processing, ELISA, quantitative PCR and immunohistochemistry

ME7 and NBH animals were perfused with heparinized saline at 12 weeks postinoculation and at 1, 2, 4, and 8 hours post-LPS/saline for enzyme-linked immunosorbent assay (ELISA) (Cunningham et al., 2007) and polymerase chain reaction (PCR). We have previously published methods for ribonucleic acid (RNA) isolation, complementary DNA (cDNA) synthesis, and quantitative PCR, as well as primer and probe sequences for many genes examined here (Cunningham et al., 2005, 2007, 2009a, 2009b; Palin et al., 2008). Further primer and probe sequences are shown in Table 1. Additional animals were perfused with 10% formalin and paraffin wax-embedded for immunohistochemistry. Sections were labeled with antibodies against synaptophysin (Sy38, Millipore, Dublin, IRL), IBA-1 (Abcam, Cambridge, United Kingdom), and IL-1β (PeproTech, London, United Kingdom) as described in previous publications (Cunningham et al., 2003, 2005, 2009a). Briefly, nonspecific peroxidase activity was eliminated by incubating sections in 1 mL H_2_O_2_/100 mL methanol (1% H_2_O_2_) for 10 minutes before antigen retrieval was carried out by microwaving in citrate buffer (pH 6) for 2 × 5 minutes (IBA-1, IL-1β) or by heating in boric acid (pH 9) for 30 minutes at 60 degrees (Sy38). After washing, sections were blocked with normal sera and incubated overnight with primary antibodies at 1/200 (IBA-1), 1/50 (IL-1β) and 1/2000 (Sy38). Labeling was completed using the appropriate biotinylated secondary antibody, ABC complex and diaminobenzidine (DAB) as chromagen before hematoxylin counterstaining. In the case of Sy38, nickel DAB was used in the place of DAB to produce a more intense, black stain and these slides were not counterstained.

### 2.6 Statistical analyses

Behavioral data were compared by repeated measures analysis of variance (ANOVA) with Bonferroni posthoc tests performed after any significant main effect. Significant interactions were further investigated with analysis of simple main effects. Percentage correct trials on Y-maze acquisition were compared by repeated measures ANOVA to assess acquisition across 2 blocks of trials. Comparisons of transcription in ME7 versus NBH animals were performed by Student *t* test. Analysis of temporal patterns of gene transcription was assessed by 2-way ANOVA comparing ME7+LPS with NBH+LPS with posthoc tests at selected time points.

## 3 Results

### 3.1 The primed hippocampus: synaptic loss and inflammatory receptors

**Fig. 1:**
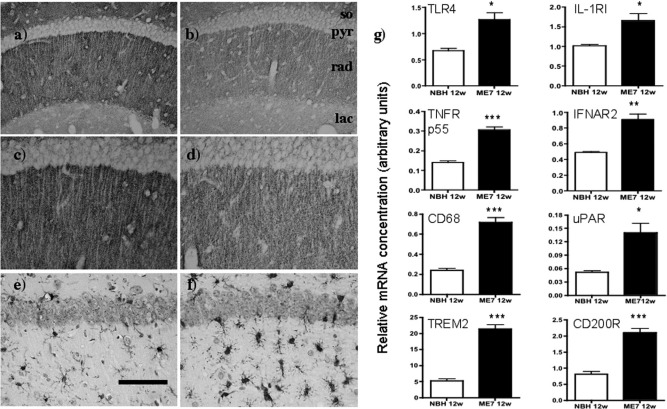
Hippocampal vulnerability: synaptic loss and inflammatory priming. Presynaptic terminals in the stratum oriens (so), stratum radiatum (rad) and stratum lacunosum moleculare (lac) of the hippocampus, visualized by immunostaining with sy38 anti-synaptophysin in NBH (a and c, ×20 and ×40, respectively) and ME7 animals at 12 weeks postinoculation (b and d, ×20 and ×40, respectively). Increased microglial numbers and activation state in the same region of NBH (e) and ME7 animals (f), labeled using anti-IBA-1 antibody against microglia and visualized at ×20. (g) Hippocampal expression of the inflammatory receptors TLR4, IL-1R1, TNFR p55, and IFNAR2, microglial CD68 and uPAR and the myeloid-restricted anti-inflammatory markers TREM2 and CD200R in ME7 and NBH animals at 12 weeks. Significant differences by Student *t* test are denoted by * (*p* < 0.05), ** (*p* = 0.0027), and *** (*p* < 0.001). *n* = 9, ME7; and *n* = 4, NBH. Scale bar = 100 μm in a and b and 50 μm in c-f.

We assessed synaptic loss in the hippocampus of ME7 animals. Presynaptic terminals are extremely numerous in the CA1 and when labeled by anti-synaptophysin antibody the strata appear as distinct layers of granular staining of relatively uniform density. In the stratum radiatum of normal animals (rad) the dendrites of the CA1 pyramidal neurons (pyr) can be seen as fine lines that remain unstained by this antibody (Fig. 1a and c). ME7 animals, at 12 weeks postinoculation, showed significant synaptic loss in the stratum radiatum of the hippocampal CA1 pyramidal cell layer compared with NBH animals at the same stage. This is most easily seen as a decreased density of staining in the stratum radiatum (rad) compared with relatively unchanged levels in the stratum lacunosum moleculare (lac) (Fig. 1a–d). The stratum radiatum also shows significantly increased microglial activation, as visualized by IBA-1 labeling (Fig. 1e and f). These IBA-1-positive cells are more numerous than in NBH animals and also show a more condensed, less ramified morphology.

We examined messenger RNA (mRNA) levels for a number of inflammatory receptors that might underpin elevated responses to the local or systemic synthesis of inflammatory mediators. The receptors toll-like receptor 4 (TLR4, *p* < 0.05), interleukin-1 receptor, type I (IL-1R1, *p* < 0.05), tumor necrosis factor receptor (TNFR, *p* < 0.0001) p55 and interferon receptor 2 (IFNAR2, *p* < 0.005) all showed significant elevation in ME7 animals compared with NBH animals when compared by Student *t* tests (Fig. 1g). That microglia are activated is confirmed by the upregulation of the phagocytic marker CD68 (*p* < 0.0001) and the microglial marker urokinase plasminogen activator receptor (uPAR, *p* < 0.05) (Cunningham et al., 2009b). That the degree of this activation is limited was indicated by the increased expression of the myeloid-specific anti-inflammatory markers triggering receptor expressed on myeloid cells 2 (TREM2) and CD200 receptor (CD200R) (*p* < 0.0001). Thus, the hippocampus is primed by loss of synapses and increased microglial activation.

### 3.2 Acute and transient working memory deficit

ME7 and NBH animals were trained on a novel paddling alternation T-maze task for 10 blocks of 10 trials. By the end of training (24 hours before LPS or saline challenge) all animals had reached a steady baseline performance at or above 80% correct choices. Student *t* test of all ME7 versus all NBH revealed that animals in the early stages of ME7-associated “dementia” (12 weeks) are not impaired with respect to controls (*p* = 0.6976) while animals at 18 weeks postinoculation are significantly slower to learn the task (Supplementary data, Figure S1).

**Fig. 2:**
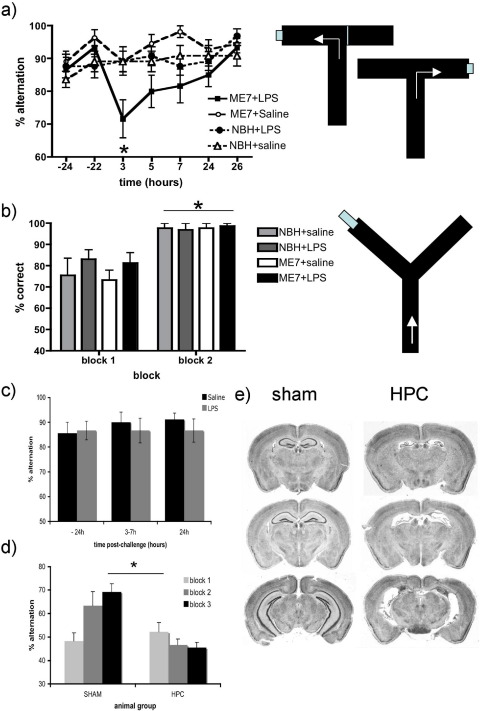
LPS-induced working memory deficits and validation of T-maze alternation. (a) Performance of ME7+LPS (*n* = 12), ME7+saline (*n* = 11), NBH+LPS (*n* = 13) and NBH+saline (*n* = 11) animals in the novel T-maze alternation task at baseline (−22, −24 hours), posttreatment with saline or LPS (3, 5, 7 hours) and upon recovery (24, 26 hours). Schematic representation of this T-maze is shown on right, illustrating forced entry to 1 arm on the sample run and alternation on the choice run. Simple main effects analysis revealed an effect of LPS specifically in the ME7 group and this is denoted by * (*p* < 0.001). (b) Performance of similar groups in the egocentric Y-maze (shown on right), 3 hours posttreatment, assessed as 2 blocks of 6 trials. Repeated measures analysis of variance (ANOVA) revealed no effect of treatment, but a main effect of block (denoted as *, *p* < 0.0001). *n* = 15, ME7+LPS; *n* = 13, NBH+LPS; *n* = 9, ME7+saline, and NBH+saline. (c) Performance of the T-maze alternation spatial working memory task (after training: 10 blocks of 10 trials) in normal C57BL6 mice treated with LPS 200 μg/kg (*n* = 9) or sterile saline (*n* = 9). There were no effects of treatment. (d) Training of hippocampal-lesioned (*n* = 8) and sham-operated animals (*n* = 12) on the spatial working memory T-maze task. A main effect of lesion group (*p* = 0.0021) in a 2-way ANOVA analysis is denoted by *. (e) Cresyl violet-stained coronal sections from representative sham-operated and hippocampal-lesioned animals.

Systemic challenge with LPS (100 μg/kg) did not induce memory impairments in NBH animals. However the same LPS challenge induced a marked working memory deficit in ME7 animals (Fig. 2a). The memory performance of NBH and ME7 groups of animals were compared across all time points postinjection with LPS or saline (i.e., 3–26 hours) using ANOVA. Importantly, there was a significant group (NBH versus ME7) by drug treatment (saline versus LPS) interaction (*F* = 9.94, *df* 1,43, *p* < 0.005). Subsequent analysis of simple main effects confirmed that there was a significant effect of LPS treatment in the ME7 group (*F* = 17.67, *df* 1,43, *p* < 0.001), but not in the NBH group (*F* < 1, *p* > 0.80). Furthermore, there was a significant difference between the groups under the effects of LPS treatment (*F* = 10.37, *df* 1,43, *p* < 0.005), but not after a saline injection (*F* = 1.70, *df* 1,43, *p* = 0.20). As with most cases of delirium, this deficit had an acute onset and was transient, consistent with a pharmacological rather than pathological disturbance.

### 3.3 Specificity of working maze deficits

Because LPS causes exaggerated sickness behavior in prion-diseased mice with respect to NBH animals (Combrinck et al., 2002), it was important to verify that any cognitive change observed was not attributable to some LPS-induced aspect of sickness that produced a nonspecific effect on performance. Parallel groups of animals were tested in a reference memory Y-maze task, similar in its motor and motivational components to the T-maze. NBH or ME7 animals, treated with LPS or saline, showed equivalent learning (Fig. 2b), and probe trials showed that correct responding in this Y-maze task was not dependent on extramaze cues (i.e., the task is egocentrically solved). Repeated measures ANOVA showed no difference between any of the experimental groups, i.e., there was a main effect of block (*F* = 27.01, *df* 1,43, *p* < 0.0001), but no effect of treatment (*F* = 0.34, *df* 3,43, *p* = 0.79) and no interaction of group and block (*F* = 0.31, *df* 3,43, *p* = 0.8187). Thus LPS does not impair learning of this task in either ME7 or NBH animals.

We also examined the effect of LPS on T-maze working memory at 200 μg/kg to assess whether exaggerated inflammation alone was sufficient to induce working memory deficits. Naive animals were treated i.p. with saline or LPS and tested in the maze between 3 and 7 hours. Alternation in the T-maze was maintained above 85% in both animal groups (Fig. 2c). Two-way ANOVA revealed no effect of treatment (*F* = 0.25, *df* 1,16, *p* = 0.62) or interaction of treatment and time (*F* = 0.38, *df* 2,32, *p* = 0.68). Therefore LPS does not affect working memory in normal animals even at the higher dose of 200 μg/kg.

Hippocampally-lesioned animals (HPC) and sham controls were placed in the T-maze for blocks of 10 trials on 3 consecutive days (*n* = 12 sham and 8 HPC; Fig. 2d). Sham-operated animals show daily increases in percentage alternation from initial chance levels while HPC animals fail to improve their performance over 3 days of training. Two-way ANOVA revealed a main effect of treatment (*F* = 12.8, *df* 1,18, *p* = 0.0021) and a significant interaction of treatment and trial block (*F* = 4.39, *df* 2,36, *p* = 0.0197). The task is therefore hippocampus-dependent. Typical hippocampal lesions are shown in Fig. 2e.

### 3.4 Time course analysis of systemic and CNS inflammation

**Fig. 3:**
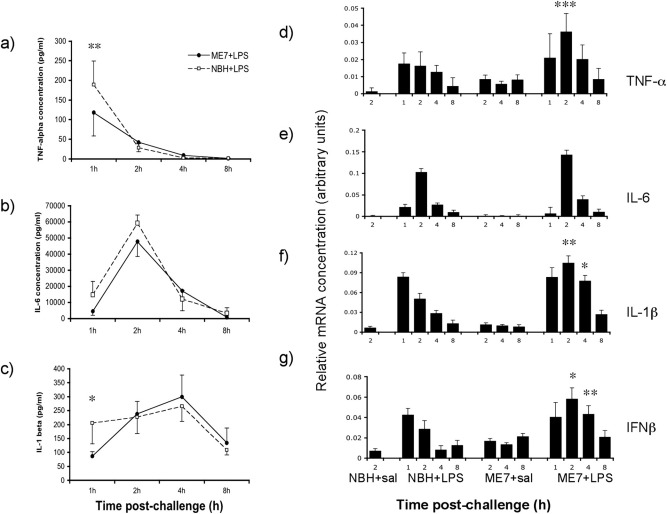
Comparison of systemic and hippocampal cytokine responses. Plasma was prepared from whole blood of NBH and ME7 animals 1, 2, 4, or 8 hours posttreatment with LPS or saline and samples were analyzed for (a) TNF-α, (b) IL-6, and (c) IL-1β protein levels. Two-way analysis of variance (ANOVA) of NBH+LPS versus ME7+LPS revealed no effect of disease for any cytokine (*F* ≤ 2.22, *df*, 1,7, *p* ≥ 0.18). There were significant interactions of treatment and time and significant pair-wise comparisons by Bonferroni posthoc are denoted by * (*p* < 0.05) or ** (*p* < 0.01). NBH+LPS (*n* = 4) and ME7+LPS (*n* = 5). Saline-treated groups did not show detectable levels of these cytokines (*n* = 4–5). (d–g) CNS cytokine transcription was assessed at the same time points by quantitative PCR. All genes were elevated after LPS treatment. ME7+LPS animals were compared with NBH+LPS group by repeated measures, 2-way ANOVA. Statistical significance by Bonferroni posthoc tests after a significant main effect is denoted by * (*p* < 0.05), ** (*p* < 0.01), or *** (*p* < 0.001). *n* = 5 for all ME7 groups and *n* = 4 for all NBH groups.

Systemic challenge with LPS (100 μg/kg) resulted in robust increases in circulating cytokines. Circulating TNF-α, IL-1β and IL-6 were below reliable detection levels in all saline-treated animals, indicating that neurodegeneration per se does not induce systemic cytokines. LPS induced an increase in TNF-α at 1 hour postinjection, with levels decreasing considerably thereafter (Fig. 3a), while IL-6 peaked at 2 hours and decreased markedly thereafter (Fig. 3b) and IL-1β was induced at more modest levels, rising gradually up to 4 hours and decreasing by 8 hours (Fig. 3c). LPS had similar effects in NBH and ME7 animals. Two-way ANOVA revealed that there was no main effect of group for any cytokine (NBH+LPS versus ME7+LPS, *F* ≤ 2.22, *df*, 1,7, *p* ≥ 0.18) but there were interactions between treatment and time post-LPS for all 3 cytokines (*F* ≥ 3.76, *df* 3,21, *p* < 0.05). Bonferroni posthoc tests revealed that ME7+LPS was actually significantly lower than NBH+LPS for both TNF-α (*p* < 0.01) and IL-1β (*p* < 0.05) at 1 hour. Thus across the full temporal profile of the major proinflammatory cytokines, ME7 animals do not show elevated systemic inflammation with respect to NBH animals similarly challenged.

Conversely, there were differences in the temporal profile of cytokine transcription in the hippocampus of LPS-challenged ME7 animals compared with LPS-challenged NBH animals (Fig. 3d–g). LPS induced equivalent transcription of cytokines in ME7 and NBH animals at 1 hour, but at 2 hours postchallenge, for all transcripts except IL-6, levels decreased in NBH+LPS animals but increased further in ME7+LPS animals. Repeated measures ANOVA analysis of ME7+LPS versus NBH+LPS, using treatment and time post-LPS as factors, revealed main effects of treatment for IL-1β, TNF-α, and IFN-β (*F* = 9.94, 10 and 7.75 respectively, *df* 1,7 *p* < 0.05 for all analyses). There was also an interaction of time and treatment for IL-1β, TNF-α, and IFN-β (*F* = 4.59, 3.13 and 4.04 respectively, *df* 3,21, *p* < 0.05). Bonferroni posthoc tests revealed that TNF-α (*p* < 0.001), IL-1β (*p* < 0.01), and IFN-β (*p* < 0.05) were significantly higher in ME7+LPS than in NBH+LPS animals at 2 hours and IL-1β (*p* < 0.05) and IFN-β (*p* < 0.01) remained so at 4 hours, but no differences were present for any cytokine at 1 hour (*p* > 0.05).

### 3.5 Altered microglial phenotype in the absence of morphological change

**Fig. 4:**
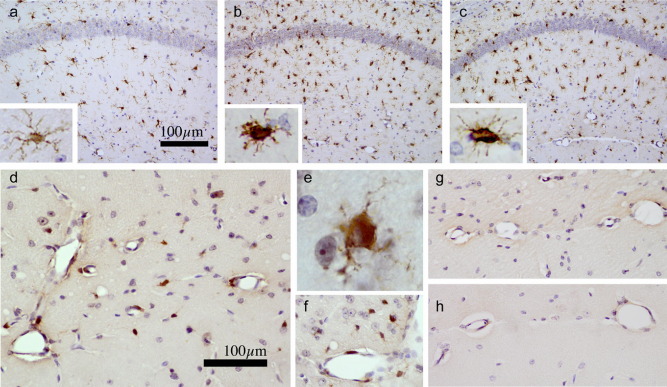
Microglial priming and further activation. IBA-1 immunolabelling has been used to demonstrate increased numbers of microglia and morphological changes in the hippocampus of ME7 animals (b) and ME7+LPS animals (c) compared with NBH animals (a) photographed at ×20 magnification. Morphological changes can be seen in insets (a–c) (×100). Immunostaining with anti-IL-1β antibody, photographed at ×40 magnification, shows perivascular and parenchymal IL-1β-positive cells at 3 hours post-LPS in the hippocampus (d) and the thalamus (f) of ME7+LPS animals, with morphology indicative of microglial cells (e; ×100). IL-1β was not detected in ME7+saline (g) or in NBH+LPS (h). Scale bar (a) represents 100 μm in pictures a, b, and c, and 20 μm in the insets. Scale bar (d) represents 100 μm in (d) and 125 μm in (f, g, and h) and 12.5 μm in (e).

Histological sections showed microglial priming in the hippocampus of ME7 animals as evidenced by increased IBA-1 positive cells with condensed, less ramified, morphological appearance in this region (Fig. 4b and c). We describe these microglia as primed rather than activated because they show this characteristic morphological appearance of activation (Fig. 4b) but are negative for IL-1β (Fig. 4g). LPS induces the synthesis of IL-1β by perivascular and proximal parenchymal microglial cells in the hippocampus and thalamus of ME7 animals (Fig. 4d–f) without inducing significant morphological changes (Fig. 4b and c insets). IL-1β is not detectable either in ME7 animals challenged with saline or in NBH challenged with LPS (Fig. 4g and h).

### 3.6 Responsive genes

**Figure S2:**
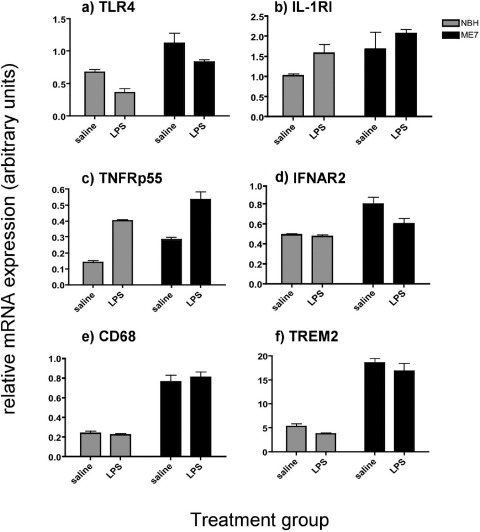
Effect of LPS or saline on mRNA expression of inflammatory receptors in ME7 and NBH animals as assessed by quantitative PCR. Transcripts a) TLR4, b) IL-1RI, c) TNFRp55, d) IFNAR2, e) CD68 and f) TREM2 were assessed 4 hours post intraperitoneal challenge with LPS (100 μg/kg) or sterile saline. Data are shown as mean ± SEM and data were analysed by two-way ANOVA using disease (ME7 vs NBH) and treatment (LPS vs saline) as factors for main effects. Results of statistical analysis are presented in full in the text. n = 5 for both ME7 groups and n = 4 for both NBH groups.

**Fig. 5:**
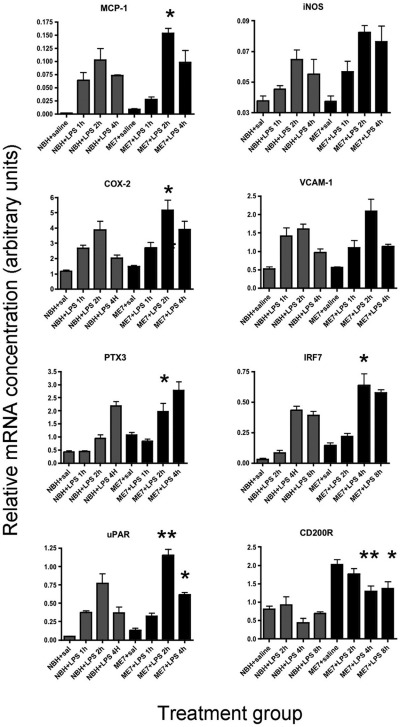
Expression of transcripts for downstream or effector molecules. Expression of microglial (uPAR, CD200R, COX-2), endothelial (VCAM-1, COX-2), NF-kB-dependent (iNOS, MCP-1, PTX3, COX-2, VCAM-1), and stat 1/2 dependent (IRF7) transcripts was examined at 1, 2, 4, and 8 hours postchallenge with LPS in NBH and ME7 animals and compared with ME7 or NBH animals treated with saline (4 hours). ME7+LPS and NBH+LPS were compared by 2-way analysis of variance (ANOVA) with treatment and time post-LPS as factors. Main effects are described in the main text. Significant differences, by Bonferroni posthoc tests, between ME7+LPS and corresponding time point in NBH+LPS, are denoted by * (*p* < 0.05), and ** (*p* < 0.01).

We examined microglial markers and genes downstream of both NF-κB (LPS, IL-1β, TNF-α signaling) and Stat 1/2 (Type I interferon signaling) activation to confirm the activity (and hence protein expression) of these inflammatory cytokines. A number of these responsive genes were found to be acutely and highly upregulated in animals treated with LPS and these are illustrated in Fig. 5. Further LPS-induced changes in expression of the inflammatory receptors assessed in NBH and ME7 in Fig. 1 have also been assessed but these are less dynamically altered by LPS and are shown in supplementary data (Fig. S2).

The genes for monocyte chemoattractant protein 1 (MCP-1) and inducible nitric oxide synthase (iNOS) both showed elevation at 1 hour and peaked at 2 hours (Fig. 5a and b). Repeated measures ANOVA comparing NBH+LPS versus ME7+LPS animals revealed no main effect of treatment for MCP-1, but an interaction of treatment and time (*F* = 4.4, *df* 3,21, *p* = 0.0149) with a significant difference at 2 hours by Bonferroni posthoc test (*p* < 0.05). For iNOS there was a main effect of treatment (*F* = 8.68, *df* 1,7, *p* = 0.0215).

Cox-2, which can be induced in both endothelial and microglial cells, showed marked expression that was greater in the ME7+LPS group than in NBH+LPS mice, but only at later time points (Fig. 5c, main effect of treatment: *F* = 9.16, *df* 1,7, *p* = 0.0192). The endothelial marker VCAM-1 was significantly induced at 1 hour, but this was not significantly different between ME7+LPS and NBH+LPS groups: there was no effect of treatment (*F* = 1.46, *df* 1,6, *p* = 0.2720) and no interaction between time and treatment (*F* = 1.35, *df* 3,18, *p* = 0.2905) (Fig. 5d).

Conversely, NF-κB-dependent PTX3 (Li et al., 2002) did not begin to show elevation until 2 hours postchallenge and peaked at 4 hours (Fig. 5e). This suggests that its transcription is downstream of proinflammatory cytokines IL-1β and TNF-α because it does not show the rapid induction of other LPS-induced genes assessed. There was a main effect of treatment (*F* = 27.32, *df* 1,7, *p* = 0.0012), with a significant post-hoc comparison at 2 hours (*p* < 0.01). Similarly the interferon-induced, stat 1/2-dependent transcript IRF7 (Doyle et al., 2002) was clearly elevated at 4 hours but not before this (Fig. 5f), consistent with its known induction downstream of interferons α/β. There was a main effect of treatment (*F* = 36.23, 1,6, *p* = 0.0009) with a significant posthoc comparison at 4 hours (*p* < 0.05).

Altered microglial activation status induced by systemic challenge was indicated by changes in myeloid-restricted gene transcription: uPAR peaked at 2 hours and was higher in ME7+LPS than in NBH+LPS (Fig. 5g). There was a significant effect of treatment (*F* = 19.44, *df* 1,7, *p* = 0.0031), an interaction of time and treatment (*F* = 3.94, *df* 3,21, *p* = 0.0225) and a significant posthoc difference at 2 hours (*p* < 0.001). The mRNA levels of the myeloid-restricted CD200R were markedly higher in ME7 animals than in NBH and decreased with time post-LPS. There were main effects of treatment (*F* = 61.89, *df* 1,25, *p* < 0.0001) and of time (*F* = 5.23, *df* 3,25, *p* < 0.01). CD200R is significantly decreased at 4 (*p* < 0.01, Bonferroni posthoc test; Fig. 5h) in ME7+LPS with respect to saline controls.

## 4 Discussion

In the current study we have shown for the first time that acute systemic inflammation at clinically relevant levels induces working memory deficits in animals early in the progression of disease in a model of dementia, but does not cause similar impairments in normal animals treated with the same or greater doses of LPS. We have demonstrated that this working memory deficit is acute and transient: these mice perform well on the task before LPS challenge, make more errors under the influence of LPS, and return to good performance as the inflammatory event resolves. This is distinct from previous reference learning deficits described by us in this model (Cunningham et al., 2009a) and from deficits in a serial spatial reversal task in aged mice treated with LPS (Chen et al., 2008). The deficits are associated with pre-existing hippocampal synaptic loss and with microglial priming that includes elevated expression of a number of inflammatory receptors, thus priming the brain to subsequent inflammatory insults. Despite equivalent systemic inflammatory responses, ME7 animals treated with LPS showed higher hippocampal transcription of proinflammatory cytokine genes than normal animals similarly treated.

### 4.1 Inflammatory mechanisms of cognitive dysfunction

Systemic inflammation can produce affective and mild cognitive changes in healthy individuals (Cohen et al., 2003; Krabbe et al., 2005; Reichenberg et al., 2001) and in severe cases (i.e., sepsis/critical illness) this can trigger delirium (Gunther et al., 2008). However, even mild to moderate systemic inflammation can precipitate delirium in aged and demented individuals. Immunotherapy with cytokines can induce delirium (IL-2, IFN) (Adams et al., 1988; Lerner et al., 1999; van Steijn et al., 2001) and increased systemic cytokines and acute phase proteins (IL-6, IL-8, CRP) are associated with delirium post hip fracture (Beloosesky et al., 2007; de Rooij et al., 2007; van Munster et al., 2008). Significantly, delirium was associated with pre-existing cognitive impairment in these hip fracture studies. Thus, while elevated systemic cytokines, in particular IL-6, are clearly a risk factor for delirium, and severity of acute insult is a predictor of delirium generally (Noimark, 2009), prior cognitive impairment is a crucial cofactor and thus pre-existing pathology is likely to be the key risk factor. In the current model we have made systemic challenges when animals already show synaptic loss and are on the cusp of the appearance of impairments in cognition (Cunningham et al., 2009a), and this model system thus allows detailed study of the interaction between systemic inflammation and prior degenerative changes.

Prior studies have shown that systemic infection with *E. coli* or treatment with LPS (Pugh et al., 1998) produces impaired contextual fear conditioning in rodents and this is more robustly impaired in aged animals (Barrientos et al., 2006). These impairments (Hein et al., 2007) and others in spatial reference memory can be replicated by making direct intrahippocampal (Matsumoto et al., 2004) but not systemic (Thomson and Sutherland, 2005, 2006) challenges with IL-1β, and those effects are dependent on prostaglandins. In a more difficult serial spatial reversal task, in young mice, LPS can produce IL-6-dependent impairments (Sparkman et al., 2006) but those studies showing working memory changes in aged versus younger animals have not yet defined key molecules in this process (Chen et al., 2008). The primed CNS/microglial response (Cunningham et al., 2005; Perry et al., 2007) has been implicated in many of these studies (Barrientos et al., 2006; Chen et al., 2008; Godbout et al., 2005) but the nature of this priming remains little investigated. We originally demonstrated that microglia proximal to existing CNS pathology (i.e., synaptic and neuronal loss and amyloid deposition) show morphological evidence of activation but do not synthesize detectable IL-1β protein, but upon systemic or central LPS challenges they display clear microglial IL-1β protein synthesis (Cunningham et al., 2005). We proposed that this primed response is not seen systemically because peripheral macrophages are not proximal to pathological changes such as those proximal to CNS microglia and indeed across the entire time course of LPS-induced systemic cytokine production, systemic levels are never higher in ME7 animals than in NBH animals. That these cytokines are initially slightly higher in NBH animals than in ME7 may be explained by the reported elevated hypothalamic pituitary adrenal axis activation in prion-diseased rodents (Voigtlander et al., 2006).

With respect to a possible molecular basis for the exaggerated CNS responses to systemic inflammation we have shown here that a number of key receptors for local responses to LPS and inflammatory cytokines are elevated in the ME7 hippocampus (TLR4, IL-1RI, TNFR p55, IFNAR2), and this may be significant in the priming of this region to inflammatory insults. Further experiments will be required to examine the functional significance of these transcriptional increases in inflammatory receptors.

The induction of cytokine transcripts in the current study is equivalent in NBH and ME7 animals at 1 hour post-LPS but levels increase at 2 hours in the ME7+LPS group, while decreasing in NBH+LPS animals. The time course data are consistent with initially equivalent transcription in the endothelial cells followed by induction of these transcripts in the primed microglia of ME7+LPS animals but significantly less transcription in NBH+LPS microglia. Many transcripts seem likely to be synthesized initially by the endothelial cell layer: iNOS and MCP-1 show induction at 1 hour, similar to known endothelial products VCAM-1 and Cox-2. Early expression of iNOS and MCP-1 cannot be explained by NF-κB-dependent transcription downstream of CNS cytokine expression. Conversely, the time course of expression indicates that PTX3 (Li et al., 2002) is transcribed downstream of IL-1β/TNF-α and that IRF7 is transcribed downstream of IFN-α/β (Honda and Taniguchi, 2006) and therefore these transcripts act as surrogate markers of the activity of IL-1β/TNF-α and IFN-α/β respectively. NF-κB induction is necessary for the transcription of many of these genes and may be the result of direct LPS signaling at the endothelium. There is considerable evidence that LPS can directly activate the endothelium in inducing CNS responses (Chakravarty and Herkenham, 2005; Gosselin and Rivest, 2008) and it is detectable in the blood within a short time of i.p. injection (Teeling, unpublished). Moreover, the rapid CNS induction of IFN-β is dependent on IRF3 (Honda and Taniguchi, 2006) and is thus induced by LPS but not by IL-1β. Thus, the current data suggest LPS signaling directly at the endothelium. While circulating inflammatory mediators certainly can induce CNS cytokines further work is required to implicate these and other inflammatory molecules specifically in the blood, at the endothelium, or in the brain parenchyma.

### 4.2 Predisposition: synaptic loss and cognitive reserve

The interaction between systemic inflammation and prior degenerative pathology in a particular region provides a conceptual framework by which we may interrogate aspects of episodes of delirium induced by systemic inflammation during dementia. The prediction, in the current study, of dysfunction of spatial working memory, as described in rodents by Olton (Olton and Feustle, 1981), was based on prior pathology in the hippocampus. This pathology is composed of synaptic terminal loss in the stratum radiatum of CA1 with accompanying microglial activation. The vulnerability of hippocampal function in this model may be caused by a loss of “cognitive reserve”. That is to say that there may be a threshold below which hippocampal synaptic activity must drop before dysfunction is observed on this working memory task. The current data are consistent with the hypothesis that disease lowers presynaptic input toward this hypothetical activity threshold and that LPS precipitates a drop in synaptic activity below the threshold and working memory deficits then appear. It is significant that even 200 μg/kg LPS did not induce working memory deficits in normal animals, suggesting that synaptic loss may be more important than microglial priming as a predisposing factor, although obviously exaggerated inflammation also increases the likelihood of impairments. Given that the cognitive changes observed were acute onset and relatively rapidly reversed upon the resolution of inflammation, we propose that LPS induced acute neurochemical rather than pathological changes in synaptic activity. It is known that inflammatory mediators such as IL-1β can affect electrophysiological aspects of synaptic function (Kelly et al., 2001) and neurotransmitter release (Taepavarapruk and Song, 2010). It should be noted that LPS may also cause pathological changes to presynaptic terminals but we propose that these are more likely to underpin long term cognitive decline, an acknowledged consequence of delirium. We have previously shown that a single systemic LPS challenge can accelerate the progression of the ME7 model of prion disease (Cunningham et al., 2009a) and altered postsynaptic architecture has also been reported after LPS challenge in aged rodents (Richwine et al., 2008). In short, we propose that microglial priming and synaptic loss constitute significant risk factors for delirium, and mild systemic inflammation constitutes a trigger that induces acute cognitive deficits only when these risk factors are present.

### 4.3 Can we model delirium in mice?

We propose that these findings are relevant to delirium during dementia. However, we do not suggest that delirium in humans is caused by selective hippocampal dysfunction. Rather, we suggest that systemic inflammation can target particular brain regions on the basis of existing pathology in those regions and that the current model allows us to study these interactions.

Delirium is a heterogeneous syndrome comprising impairments in attention, orientation, memory, level of arousal, perception and affect, and psychotic disturbances. Detecting this full spectrum of dysfunction in a nonverbal animal such as a mouse is clearly not possible. Thus, we have focused on a number of key features that are potentially analogous to some core cognitive and temporal features of delirium. Firstly episodes are of acute onset and transient and can be distinguished from chronic cognitive impairments. Secondly, the interaction of systemic inflammation and existing (Elie et al., 1998; Lerner et al., 1997) or incipient (Rahkonen et al., 2001) dementia is 1 very common multifactorial etiological route to clinical delirium and is mimicked in a biologically relevant way in the current study. Thirdly, there are similarities in the nature of the cognitive deficits observed in the T-maze and allocentric Y-maze and some of those in human delirium: ICD-10 diagnostic criteria include “impairment of recent memory, … disorientation for time as well as, in more severe cases, for place” (World Health Organization, 1992). Acquisition of the allocentric Y-maze task in a novel environment is impaired in ME7+LPS animals (Cunningham et al., 2009a) but retention performance is not impaired by LPS when the environment has become familiar after prior training (Cunningham, unpublished data). Adding the temporal components required for performance in the working memory T-maze reveals acute and transient dysfunction even in a familiar environment. Thus LPS-induced impairments become apparent when processing and retention of novel, trial-specific information, for just 25 seconds is required to accurately perform the task. Patients with delirium perform particularly badly relative to patients with dementia on tasks requiring retention and processing of novel, trial-specific information over similar periods (Hart et al., 1996, 1997).

Given the hypothesis that prior pathology is crucial, it is significant that learning in the egocentric Y-maze, in which correct responding does not require attention to extramaze visual cues, was not affected by LPS in ME7 animals (Fig. 2b). The striatum contributes more significantly to the performance of egocentric tasks, than does the hippocampus (Cook and Kesner, 1988; Mitchell and Hall, 1988; White, 1997) and does not show significant prior pathology in the ME7 model. We predict that more global pathology, such as that observed in Alzheimer's disease (deKosky and Scheff, 1990), would predict more global cognitive dysfunction upon systemic inflammatory insult. Therefore the current model, with good reason, predicts only a subset of delirium symptoms. Finally, it is worth noting that spatial working memory alternation tasks, analogous to those used here, are also acetylcholine dependent (Bontempi et al., 2003; Givens and Olton, 1995) and prior studies have modeled delirium by pharmacological disruption of cholinergic activity (Leavitt et al., 1994; Tamura et al., 2006). Thus the current hypothesis and hypocholinergic accounts of delirium are not mutually exclusive.

### 4.4 Concluding remarks

Addressing our limited understanding of delirium pathophysiology is extremely important both from the point of view of prevention or treatment of the acute episode, but also as a means of minimizing the recognized long term consequences of such episodes. We have now shown that animals with chronic neurodegeneration, upon systemic inflammatory challenge, show acute and transient cognitive decrements comparable to those described in delirium. Because this model also shows acceleration of disease post-LPS (Cunningham et al., 2009a), it is predicted that detailed examination of the mechanism of inflammatory exacerbation that triggers the acute cognitive changes observed in this model will have implications for our understanding of delirium and the role of inflammation in neurodegenerative disease. These mechanisms are under investigation in our laboratory.

## Disclosure statement

All animal procedures were done in strict accordance with UK Home Office license and Irish Department of Health regulations.

The authors disclose no potential or actual conflicts of interest, financial or otherwise.

## Figures and Tables

**Table 1:** Mouse Taqman primer and probe sequences

αTarget	Oligonucleotide	Sequence	Amplicon size (bp)
CD68	Forward primer	5′-CAAGGTCCAGGGAGGTTGTG-3′	75
	Reverse primer	5′-CCAAAGGTAAGCTGTCCATAAGGA-3′	
	Probe	5′-CGGTACCCATCCCCACCTGTCTCTCTC-3′	
uPAR	Forward primer	5′-TGCAATGCCGCTATCCTACA-3′	116
	Reverse primer	5′-TGGGCATCCGGGAAGACT-3′	
	Probe	5′-CCCTCCAGAGCACAGAAAGGAGCTTGAA-3′	
TLR4	Forward primer	5′-GGCTCCTGGCTAGGACTCTGA-3′	114
	Reverse primer	5′-TCTGATCCATGCATTGGTAGGT-3′	
	Probe	5′-CATGGCACTGTTCTTCTCCTGCCTGA-3′	
IL-1R1	Forward primer	5′-GCAATATCCGGTCACACGAGTA-3′	117
	Reverse primer	5′-ATCATTGATCCTGGGTCAGCTT-3′	
	Probe	5′-TCCTGAGCCCTCGGAATGAGACGATC-3′	
IFNAR2	Forward primer	5′-GAGAGCAGAAAAACGGACTTAAGAG-3′	98
	Reverse primer	5′-TCGCAGACACCACAAGACACA-3′	
	Probe	5′-TGCACCGTCTCTGCCGTCGG-3′	
Trem2	Forward primer	TGTGGTCAGAGGGCTGGACT	68
	Reverse primer	CTCCGGGTCCAGTGAGGA	
	Probe	CCAAGATGCTGGGCACCAACTTCAG	
IRF7	Forward primer	CGAGGAACCCTATGCAGCAT	108
	Reverse primer	TACATGATGGTCACATCCAGGAA	
	Probe	CCAGCTCTCACCGAGCGCAGC	
CD200R1	Forward primer	AGGAGGATGAAATGCAGCCTTA	80
	Reverse primer	TGCCTCCACCTTAGTCACAGTATC	
TNF R p55	Forward primer	GCTGACCCTCTGCTCTACGAA	132
	Reverse primer	GCCATCCACCACAGCATACA	
VCAM-1	Forward primer	GATGTAAAAGGAAAAGAACATAACAAGAAC	90
	Reverse primer	GATGGCAGGTATTACCAAGGAAGA	

Where probe is not included, Sybr green has been used in its place.

## Detailed methods

### Correction strategy in T maze alternation

If animals develop a side preference early in training a correction strategy can be employed. If animals display a side preference this can be broken by not allowing them to self-correct: ie instead of crossing the T from the incorrect arm to the correct arm, the entrance to the correct arm can be closed at the moment that the incorrect arm is chosen. Then animals must return to the start arm and once again have the option to choose the correct arm. This is repeated until the animal finally chooses the correct body turn when starting from the start arm. This correction strategy is only employed for animals displaying a complete side preference (ie always turning in one direction if that arm is open to them), is discontinued once their side preference is broken and is never employed within 2 days of administration of substances to examine effects on working memory.

### ELISA protocol

Briefly, the capture anti-IL-1β, IL-6, and TNF-α antibodies were all diluted 1/180 (approximately 1 μg/ml) in PBS and used to coat a 96-well maxisorb microplate overnight (Nunc; Fisher Scientific, Leicestershire) with 100 μl per well. Plates were then washed with PBS + 0.05% Tween and blocked with PBS + 1% BSA before addition of samples, serially diluted as before. Standards were prepared in the range 8-1000 pg/ml. Detection antibodies were used at 1/180 in 1% BSA/PBS and Streptavidin poly horseradish peroxidase was diluted 1:10,000 in high performance ELISA buffer. TMB and H_2_O_2_ were used as substrate and the reaction was stopped with 1M H_2_SO_4_ before reading the optical density at 450 nm with correction at 570 nm. The reliable quantification limit of all assays was 15.6 pg/ml.

### Quantitative RT-PCR

Animals challenged intraperitoneally with saline or LPS (100μg/kg) were terminally anaesthetized at 1, 2, 4 or 8 hours post-challenge and then transcardially perfused with heparinised saline. Brains were rapidly removed and hippocampi punched out, placed in eppendorf tubes, snap frozen on liquid nitrogen and stored at −80 °C until further use. Total RNA was extracted from brain samples using Qiagen RNeasy® Plus mini kits (Qiagen, Crawley, UK) according to the manufacturer's instructions. Contaminating genomic DNA was eliminated via degradation during extraction using the Qiagen RNase-free *DNase1* enzyme. Yields were determined by spectrophotometry at λ 260 nm. RNA was stored at −80 °C until cDNA synthesis and PCR assay. All equipment and reagents were supplied by Applied Biosystems (Warrington, UK). Primer and probe sequences for IL-1β, IL-6, TNF-α, IFNβ, PTX-3, iNOS, uPAR, CD68, COX-2 were as previously published (Cunningham et al., 2005, 2007, 2009, Palin 2008) while those for IL-1R1, TNFR p55, IFNAR2, TLR4, MCP-1, VCAM1, IRF7, TREM2, and CD200R were designed using the published sequences for these genes, applied to Primes Express™ software. Where possible, probes were designed to cross an intron such that they were cDNA specific. Table 1 lists the sequences for primers and probes for each assay previously unpublished. In some cases (noted below) the fluorescent DNA binding probe SYBR green has been used in place of a specific probe. All primer pairs were checked for specificity by standard reverse transcription (RT)-PCR followed by gel electrophoresis and each primer pair produced a discrete band of the expected amplicon size (supplementary information). For Taqman PCR, cDNA was generated from total RNA using a High Capacity cDNA Reverse Transcription Kit (Applied Biosystems). Two hundred nanograms of total RNA were reverse transcribed in a 10μl reaction volume. One microliter of the RT reaction (equivalent to 20 ng of RNA) was subsequently used for the PCR, as described previously (Cunningham et al., 2007).

A standard curve was constructed from total RNA isolated from mouse brain tissue 24 hours after intra-cerebral challenge with 2.5 μg LPS, which is known to up-regulate all target transcripts of interest in this study. This standard curve was constructed using a higher concentration of RNA in the reverse transcriptase reaction than that in the samples for analysis to ensure that the cytokine transcription of all experimental animal groups will fall within the range of the standard curve constructed. Serial 1 in 5 dilutions of the cDNA synthesized from brains of LPS-injected mice were made and a curve plotted of the Ct value (the cycle number at which the fluorescence of the product of transcribed gene crosses the threshold of detection) versus the log of the concentration (assigned an arbitrary value since the absolute concentration of cytokine transcripts is not known). Verification that this curve is a straight line confirms the efficiency of the PCR reaction across the entire concentration range and the equation of this line can then be used to calculate the relative concentrations of the experimental samples. Since the top standard has been assigned an arbitrary value, it follows that the calculated concentrations of all experimental samples will also have arbitrary values, but these values will be directly related to the equation of the line.

The housekeeping gene glyceraldehyde-3-phosphate dehydrogenase (GAPDH) was measured in each sample using Applied Biosystems Rodent GAPDH Taqman Kit. All PCR data were normalised to the expression of GAPDH.

## Results

### T-maze alternation in ME7 and NBH animals at 18 weeks

Disease progression in the ME7 model, at 18 weeks, involves considerable hippocampal neuronal and synaptic loss. We assessed whether animals at this stage could learn the T-maze alternation task used elsewhere in this study. Across 4 blocks of 10 trials NBH animals improved their performance markedly, while similar improvements were not made by ME7 animals. These data are shown in figure S1a. ANOVA revealed a significant effect of treatment (F = 9.56, *df* 1,18, p = 0.0063), a significant effect of time (F = 6.75. df 3,54, p = 0.0006).

NBH and ME7 animals, at 10 weeks post-inoculation were trained on the T-maze alternation task for ten blocks of ten trials over two weeks, such that they would reach stable baseline performance >80% before 12 weeks. This is shown in figure S1 (bottom panel). ME7 and NBH animals learned this task at the same rate. There was no effect of treatment (F = 0.13, *df* 1,34, p = 0.7184) but a main effect of block (F = 26.15, *df* 9,306, p < 0.0001). These data are shown in figure S1b.

## Altered expression of inflammatory receptor post-LPS

We assessed whether the panel of cell surface inflammatory receptors assessed in Figure 1 (main text) were further altered after treatment with LPS or saline. These data are shown in figure S2.

Treatment with LPS induced a decrease in TLR4 mRNA expression in both NBH and ME7 animals at 4 hours post-challenge. There was a main effect of disease (F = 23.3, df 1,14, p = 0.0003) and of treatment (F = 9.75, df 1,14, p = 0.0075) but no interaction between these factors.

IL-1RI showed a significant main effect of disease (F = 4.68, df 1,14, p = 0.048) but no significant main effect of treatment (F = 3.26, df 1,14, p = 0.0925) despite slight increases in these transcripts post-LPS. There was also no significant interaction between these factors.

LPS also increased TNFRp55 by approximately 2-fold. There were main effects of treatment (F = 70.14. df 1,13, p < 0.0001) and of disease (F = 20.42, df 1,13, p = 0.0006) for TNFRp55 but there was no interaction between these factors (p = 0.8968).

IFNAR2 was increased in ME7 with respect to NBH but was not further increased by LPS. There were main effects of disease (F = 18.21, df 1,14, p = 0.0008) but no significant effect of treatment (ie LPS: F = 4.5, df 1,14, p = 0.0523). Though LPS did appear to decrease expression of IFNAR2 in ME7 animals, there was no significant interaction between disease and treatment (F = 3.29, df 1,14, p = 0.09).

Neither CD68 nor TREM2 showed any response to LPS in either ME7 or NBH animals. There were main effects of disease in both cases (CD68: F = 103.09, df 1,13, p < 0.0001 and TREM2: F = 136.94, df 1,13, p < 0.0001) but no effect of treatments (CD68: p = 0.8190; TREM2: p = 0.1898) nor any interaction between these factors.
